# Assessing knowledge and perception of artificial intelligence in solid organ transplantation

**DOI:** 10.3389/fdgth.2026.1834577

**Published:** 2026-07-08

**Authors:** Lea Liaigre, Dorothee Lombardo, Marie-Camille Chaumais, Bruno Degano, Pierrick Bedouch, Sebastien Chanoine

**Affiliations:** 1 Pharmacy Department, Grenoble Alpes University Hospital, Grenoble, France; 2 Univ. Grenoble Alpes, TIMC UMR5525, Grenoble, France; 3 Pharmacy Department, Assistance Publique - Hôpitaux de Paris (AP-HP), Hôpital Bicêtre, Le Kremlin-Bicêtre, France; 4 INSERM UMR_S 999 “Pulmonary Hypertension: Pathophysiology and Novel Therapies”, Hospital Marie Lannelongue, Le Plessis-Robinson, France; 5 Thorax and Vascular Department, Pneumology Clinic, Univ. Grenoble Alpes, Grenoble Alpes University Hospital, Grenoble, France; 6 Univ. Grenoble Alpes, CNRS, Grenoble INP, TIMC UMR5525, Grenoble, France; 7 Univ. Grenoble Alpes, INSERM U1209, CNRS UMR 5309, Institut Pour L'Avancée des Biosciences (IAB), Grenoble, France

**Keywords:** artificial intelligence, cross-sectional study, knowledge, perception, solid organ transplantation

## Abstract

**Introduction:**

Artificial intelligence (AI) is increasingly integrated into healthcare, reshaping clinical decision-making and care pathways. In solid organ transplantation (SOT), where clinical complexity and uncertainty are high, AI holds particular promise. Yet, beyond technical performance, its successful adoption depends on how it is understood and perceived by patients and healthcare professionals (HP). To date, little is known about these perspectives in SOT. This study provides the first assessment of knowledge and perceptions of AI among patients and healthcare professionals in this field.

**Methods:**

A cross-sectional study was conducted among SOT patients and HP. Patients were recruited through associations, and HP through the French Society of Transplantation. Data were collected using a web-based questionnaire including open-ended and closed items addressing sociodemographic characteristics, declared knowledge, and perception of AI and its applications in healthcare and SOT. Thematic coding was used for qualitative data, regression models for association analyses, and reliability testing was assessed (Cronbach's α > 0.7).

**Results:**

218 patients and 92 HP participated. Patients reported low AI declared knowledge (mean 2.4/10), while HP suggested slightly higher levels (3.8/10). 140 patients had heard of AI in healthcare, and very few were aware of its use in SOT (*n* = 20). Although most HP were familiar with AI in healthcare (*n* = 71), declared knowledge of AI applications in SOT remained limited (*n* = 39). Overall, both groups expressed positive perceptions of AI in SOT, with concerns mainly related to dehumanization of care and potential errors. Patients largely viewed AI as a complement too, rather than a replacement for healthcare professionals.

**Discussion:**

As declared knowledge was associated with perceptions of AI, these findings highlight the importance of targeted educational interventions to promote informed integration of AI in SOT. This exploratory study provides valuable insights into potential facilitators and barriers to adoption, providing actionable insights to guide implementation strategies and policy development in transplant care.

## 1 Introduction

While relatively unfamiliar to the public in the early 2000s, the term artificial intelligence (AI) has transitioned from a technical concept to a widely recognized term in public discourse. Defined in 1956 by John McCarthy, the concept of AI has evolved and been refined over the years, but its objective remains the same: to use computers to understand human intelligence and solve problems more efficiently and satisfactorily than human beings (1). There are several types of AI, the most important being machine learning (2). In healthcare, its potential of applications is considerable, offering opportunities to improve the quality of patient care while improving system efficiency and reducing overall costs (3). Numerous applications are beginning to emerge in all healthcare areas, such as diagnosis, monitoring and therapy (4, 5). Solid organ transplantation (SOT) is an interesting example of a field in which AI could be applied. Four issues are beginning to be investigated: organ allocation, precision transplant pathology analysis, prediction of post-transplant survival and therapeutic pharmacological monitoring (6, 7). Several research projects are underway in this field and several concrete applications are already in place (8–10).

However, AI is the subject of many controversies, notably on the risk of job loss, data protection, ethics, and responsibilities (11). Many subjects crystallize concerns, making it even more important to better understand the perception and declared knowledge of both patients and healthcare professionals to better inform them and improve their adherence (12).

Health perception refers to an individual's subjective assessment of their health status, symptoms, and the information provided by healthcare professionals. Health declared knowledge refers to the comprehension of medical information, available treatments, and the implications of therapeutic choices. A range of validated instruments can be used to evaluate perception and declared knowledge in health, including self-assessment questionnaires, clinical interviews, and comprehension tests. These evaluations enable healthcare professionals to tailor their interventions, address misunderstandings, and optimize communication strategies, thereby promoting informed decision-making and enhancing treatment adherence.

Despite increasing integration of AI into healthcare, understanding among patients and healthcare professionals remains variable, particularly in specialized fields such as SOT. Existing trials suggested a generally positive perception (13, 14). However, none has been conducted on the application of AI in SOT, for which perceptions and declared knowledge are specific and crucial for care adherence and therefore therapeutic success of the transplantation. Indeed, stakeholders' declared knowledge and perceptions are pivotal determinants of clinical decision-making and trust in novel technologies. Understanding these dimensions is essential to anticipate barriers and facilitators for the adoption of AI tools, ensuring that implementation strategies align with users' expectations and ethical considerations. This enhances the likelihood of integrating AI effectively into transplantation care pathways, improving patient outcomes and healthcare efficiency. The objective of the study was to explore for the first time declared knowledge and perception of AI in SOT among patients and healthcare professionals through an online questionnaire.

## 2 Methods

### 2.1 Study design

A cross-sectional study was conducted in France among SOT patients and healthcare professionals (“Trial Registration: ClinicalTrials.gov NCT05932030”).

### 2.2 Objectives of the study

The primary objective of this study was to assess the level of declared knowledge and perception of SOT patients and healthcare professionals working in SOT. The primary endpoint was the qualitative and quantitative analysis of responses to open-ended and closed-ended questionnaire items, focusing on thematic representations of declared knowledge and perception. Secondary objectives were to compare the level of declared knowledge and perception between different healthcare professionals on AI, among patients according to the transplanted organ, and finally between healthcare professionals and patients. The secondary endpoints were the comparison of averages obtained for each group.

### 2.3 Survey construction

The main themes for the questionnaire were identified through a literature review on the application of AI to SOT, using PubMed, Embase, and Cochrane databases. This review guided the selection of key topics and informed the development of the questionnaire. The search strategy included a combination of free-text terms and MeSH terms related to AI, SOT, cross-sectional studies, declared knowledge, and perception. Based on the findings of this literature review, a piloted web-based questionnaire was generated and subsequently distributed through a network of patient and professional associations. The questionnaire was adapted to the two populations. As this survey-based, cross-sectional study involved no researcher–participant interaction and followed an observational design, methodological reporting adhered to the STROBE guidelines rather than interview-focused frameworks such as COREQ.

The questionnaire was divided into 24 items for patients and 26 items for professionals. It was constructed around three main sections: socio-demographic, self-assessment of declared knowledge of AI and perception of AI in general, AI healthcare applications, and AI applications in SOT, and opinion about AI applications (details available in the Supplemental Material).

### 2.4 Sampling selection

All adults who had undergone at least one SOT, no matter how long the post-transplant period, were eligible for inclusion in the study. Healthcare professionals had to work in SOT, and could be specialist physicians, surgeons, pharmacists, residents, or coordinating nurses. The exclusion criteria were: age under 18, having undergone a hematopoietic stem cell transplant, or being under guardianship or deprived of liberty. Participants were recruited using a combination of convenience and snowball sampling methods. Patients receiving care at the investigators' institutions were invited directly, while additional participants were reached through patient and healthcare professional associations (French Society of Transplantation) that disseminated the questionnaire within their respective networks. The kidney and heart transplant patient associations did not agree to distribute the questionnaire to patients.

### 2.5 Ethical approval

Participants were included based on informed non-opposition, in accordance with French regulatory requirements for category 3 observational studies and in accordance with ethical principles of the Declaration of Helsinki. Ethical approval was obtained from the Personal Information Protection Commission (PCP) Ouest IV with a reference number of 2023-A00950–45. Participation in the study was confidential and voluntary; data were pseudonymized upon collection and securely stored in accordance with the General Data Protection Regulation (GDPR). No identifying information was accessible to the research team, and participants could withdraw at any time without justification. Additional information is available in the Supplemental Material.

### 2.6 Statistical analysis

Continuous variables were expressed as means with corresponding standard deviations, such as the mean declared knowledge score out of 10. Categorical variables were described using frequencies and percentages. To assess patients' perception, a qualitative analysis of the answers to the open-ended questions was carried out (15). A thematic content analysis was conducted. Open-ended responses were analysed using a qualitative descriptive approach and were independently coded by two researchers (LL and SC) with inductive thematic analysis. Categories were discussed and refined iteratively with a third evaluator (MS) until consensus. The analysis was performed using Excel (version 2506), ensuring systematic data organization and traceability of coding decisions. Associations between professional type or organ type and continuous variables were assessed using linear regression; Pearson's Chi-squared test was used for categorical variables. The comparison of declared knowledge levels and perception between healthcare professionals and patients was adjusted for age because the age distribution was different between the two groups, with patients being older than healthcare professionals. Multiple testing was corrected using the Benjamini-Hochberg method. In case of missing data on categorical variables, they were managed by multiple imputation, assuming that the missing data mechanism was Missing At Random. Variables included in the imputation model comprised variables with missing values. Twenty imputations were generated according to Rubin's rules and the number of missing data.

To assess the internal consistency of the questionnaire, a reliability analysis was performed using Cronbach's alpha, ensuring that they reliably assess the same underlying construct (16). A Cronbach's alpha value above 0.7 was considered acceptable for internal consistency (17). The analysis was conducted separately for different sections of the questionnaire to verify the reliability of each domain (16). All statistical analyses were performed using RStudio (version 4.2.1). A *p*-value <0.05 was established as the threshold for statistical significance.

## 3 Results

A total of 218 and 92 responses were obtained for patients and healthcare professionals, respectively. A significant proportion of people interviewed did not answer the entire questionnaire (patients, *n* = 101; healthcare professionals, *n* = 43), mostly on qualitative questions. The number of missing items per patient ranged from 1 to 6. Most patients had 2 or 3 missing items (19.1% each).

### 3.1 Internal consistency

Cronbach's alpha coefficient was equal to 0.645 (95%CI: 0.574–0.713) for the assessment of declared knowledge (questions 8 to 18) and 0.461 (95%CI: 0.123–0.619) for the evaluation of patients' perception (questions 19 to 23).

It was equal to 0.728 (95%CI: 0.632–0.788) for the assessment of declared knowledge (questions 7 to 18) and 0.711 (95%CI: 0.12–0.802) for the assessment of perception among healthcare professionals (questions 19 to 23).

### 3.2 Patients

Most patients were male (56.4%, *n* = 104/218, *p* = 0.047); 18 patients did not give their gender. They were mostly over 50 years old (78.3%, *n* = 168, *p* = 0.032) and retired (40.5%, *n* = 89). The most common transplanted organ type was liver (61.5%, *n* = 134), and almost all patients had undergone a single organ transplant, for an average of 9 years. (Table 1).

**Table 1:** Characteristics of transplanted patients by organ type, with between-group statistical analysis.

Transplanted Organ	Heart (*N* = 31)	Kidney (*N* = 19)	Liver (*N* = 134)	Lung (*N* = 34)	Total (*N* = 218)	*p* value	
**Sex**	**Sex**	**Sex**	**Sex**	**Sex**	**Sex**	0.0471	0.0471
Male *n* (%)	20 (76.9%)	5 (33.3%)	64 (56.1%)	15 (51.7%)	104 (56.5%)		
**Age**	**Age**	**Age**	**Age**	**Age**	**Age**	0.0321	0.0321
18–25 years old *n* (%)	0 (0%)	0 (0%)	0 (0%)	1 (2.9%)	1 (0.5%)		
26–35 years old *n* (%)	2 (6.5%)	2 (10.5%)	3 (2.3%)	1 (2.9%)	8 (3.7%)		
36–50 years old *n* (%)	7 (22.6%)	5 (26.3%)	17 (12.9%)	10 (29.4%)	39 (18.1%)		
51–65 years old *n* (%)	17 (54.8%)	6 (31.6%)	55 (41.7%)	12 (35.3%)	90 (41.7%)		
65 years old and more *n* (%)	5 (16.1%)	6 (31.6%)	57 (43.2%)	10 (29.4%)	78 (36.1%)		
**Number of SOT**	**Number of SOT**	**Number of SOT**	**Number of SOT**	**Number of SOT**	**Number of SOT**	<0.0011	<0.0011
1, *n* (%)	29 (96.7%)	11 (57.9%)	120 (91.6%)	32 (94.1%)	192 (89.7%)		
2, *n* (%)	1 (3.3%)	8 (42.1%)	8 (6.1%)	2 (5.9%)	19 (8.9%)		
3, *n* (%)	0 (0%)	0 (0%)	2 (1.5%)	0 (0%)	2 (0.9%)		
More than 3, *n* (%)	0 (0%)	0 (0%)	1 (0.8%)	0 (0%)	1 (0.5%)		
**Year of the last SOT**	**Year of the last SOT**	**Year of the last SOT**	**Year of the last SOT**	**Year of the last SOT**	**Year of the last SOT**	0.1492	0.1492
Mean (SD)	2014.2 (7.8)	2017.1 (7.1)	2013.6 (8.2)	2016.1 (5.6)	2014.4 (7.7)		
**I know about AI**	**I know about AI**	**I know about AI**	**I know about AI**	**I know about AI**	**I know about AI**	0.2211	0.2211
Yes, n/ *n* of patients who responded to the item (%)	26/27 (96.3%)	12/14 (85.7%)	79/98 (80.6%)	21/27 (77.8%)	138/166 (83.1%)		
**Context of AI declared knowledge**	**Context of AI declared knowledge**	**Context of AI declared knowledge**	**Context of AI declared knowledge**	**Context of AI declared knowledge**	**Context of AI declared knowledge**	0.3981	0.3981
Entourage, *n* (%)	1 (3.8%)	1 (7.7%)	2 (2.3%)	3 (12%)	7 (4.6%)		
Medias, *n* (%)	18 (69.2%)	9 (69.2%)	65 (73.9%)	16 (64%)	108 (71.1%)		
Medical, *n* (%)	1 (3.8%)	2 (15.4%)	8 (9.1%)	4 (16%)	15 (9.9%)		
Professional, *n* (%)	6 (23.1%)	1 (7.7%)	13 (14.8%)	2 (8%)	22 (14.5%)		
**AI declared declared knowledge**	**AI declared declared knowledge**	**AI declared declared knowledge**	**AI declared declared knowledge**	**AI declared declared knowledge**	**AI declared declared knowledge**	0.0952	0.0952
Mean (SD)	3.3 (2)	2.5 (2.8)	2.4 (2.3)	1.8 (1.6)	2.4 (2.3)		
**I know about AI on healthcare**	**I know about AI on healthcare**	**I know about AI on healthcare**	**I know about AI on healthcare**	**I know about AI on healthcare**	**I know about AI on healthcare**	0.9761	0.9761
Yes, n/ n of patients who responded to the item (%)	10/27 (40%)	6/15 (40%)	43/98 (43.9%)	11/27 (40.7%)	70/174 (42.4%)		
**AI on healthcare declared declared knowledge**	**AI on healthcare declared declared knowledge**	**AI on healthcare declared declared knowledge**	**AI on healthcare declared declared knowledge**	**AI on healthcare declared declared knowledge**	**AI on healthcare declared declared knowledge**	0.2472	0.2472
Mean (SD)	1.9 (2.1)	1.4 (2.6)	1.3 (1.8)	0.9 (1.4)	1.3 (1.9)		
Range	0–9	0–9	0–9	0–5	0–9		
**I know about AI on SOT**	**I know about AI on SOT**	**I know about AI on SOT**	**I know about AI on SOT**	**I know about AI on SOT**	**I know about AI on SOT**	0.841	0.841
Yes, n/ n of patients who responded to the item (%)	4/27 (14.8%)	3/14 (21.4%)	7/104 (6.7%)	6/28 (21.4%)	20/173 (11.6%)		
**AI on SOT declared declared knowledge**	**AI on SOT declared declared knowledge**	**AI on SOT declared declared knowledge**	**AI on SOT declared declared knowledge**	**AI on SOT declared declared knowledge**	**AI on SOT declared declared knowledge**	0.2752	0.2752
Mean (SD)	0.9 (1.3)	0.6 (1.2)	0.4 (1)	0.4 (1.1)	0.5 (1.1)		
**Most popular applications**	**Most popular applications**	**Most popular applications**	**Most popular applications**	**Most popular applications**	**Most popular applications**	0.421	0.421
Medical imagery analysis, *n* (%)	1 (3.8%)	2 (15.4%)	13 (15.5%)	4 (16%)	20 (13.5%)		
Morbidity-mortality prediction, *n* (%)	1 (3.8%)	2 (15.4%)	20 (23.8%)	4 (16%)	27 (18.2%)		
Donor-recipient matching, *n* (%)	17 (65.4%)	9 (69.2%)	46 (54.8%)	14 (56%)	86 (58.1%)		
Therapeutic follow up, *n* (%)	7 (26.9%)	0 (0%)	5 (6%)	3 (12%)	15 (10.1%)		
**AI opinion**	**AI opinion**	**AI opinion**	**AI opinion**	**AI opinion**	**AI opinion**	0.3722	0.3722
Mean (SD)	5.8 (2)	5.9 (2.3)	6 (2.3)	5 (2.4)	5.8 (2.3)		
**Data sharing**	**Data sharing**	**Data sharing**	**Data sharing**	**Data sharing**	**Data sharing**	0.2381	0.2381
All the data, *n* (%)	12 (57.1%)	9 (75%)	50 (64.1%)	17 (77.3%)	88 (66.2%)		
Some basic data, *n* (%)	7.0 (33.3%)	0.0 (0.0%)	14.0 (17.9%)	3.0 (13.6%)	24.0 (18.0%)		
Very few data, *n* (%)	2.0 (9.5%)	3.0 (25.0%)	14.0 (17.9%)	2.0 (9.1%)	21.0 (15.8%)		
**Find out more about AI**	**Find out more about AI**	**Find out more about AI**	**Find out more about AI**	**Find out more about AI**	**Find out more about AI**	0.0661	0.0661
Yes *n* (%)	11.0 (55.0%)	11.0 (100.0%)	45.0 (64.3%)	13.0 (72.2%)	80.0 (67.2%)		

1 Pearson's Chi-squared test.

2 Linear Model ANOVA.

In cases where data was missing, this was specified for each item concerned.

### 3.3 Artificial intelligence declared knowledge

Most patients had already heard of AI (83.2%, *n* = 138), and the main source of information was the media (71.1%, *n* = 108). Their average declared level (ADL) of knowledge was very low (mean = 2.4/10). The most familiar type of AI was the conversational agent (23.4%, *n* = 17), but half of the patients did not know any type of AI. 42.4% (*n* = 140) had heard of the application of AI in healthcare, with a very low ADL of knowledge (mean = 1.3/10). A minority of patients had heard of AI in SOT (11.6%, *n* = 20), with ADL close to zero (mean = 0.5/10). (Table 1).

### 3.4 Artificial intelligence perception

Patient perceptions were moderately positive (mean = 5.8/10). Most patients felt that AI would be of benefit in improving immunosuppressant and side effect monitoring, predicting rejection, and the risk of complications (Table 1).

When asked to give 5 words that remind them of AI, 128 words were mentioned, divided into 5 categories: legal framework, fields of application, definition and techniques used, positive points, and negative points. The “positive” category was the most represented, in line with patients' positive perception of AI. The most frequent words were “help” (14.7%, *n* = 33), “robot” (12.5%, *n* = 28), “future” (12.4%, *n* = 27), and “progress” (9.8%, *n* = 22). ChatGPT was the most cited application.

The two major risks were the potential for errors (12.5%, *n* = 28) and the dehumanization of medicine (9%, *n* = 20). Some patients identified no risk at all in using AI.

The opportunities perceived were the time-saving that AI could bring, particularly on the pre-transplant pathway. They perceived AI as more accurate than conventional medicine. A significant proportion of patients had no opinion. The most requested application of AI to SOT by patients was donor-recipient matching (58.1%, *n* = 86). The use of AI to predict the risk of morbidity and mortality was ranked last.

Most patients (66.2%, *n* = 88) were willing to share all their health data for use by AI in their care and expressed interest in learning more about AI after completing the questionnaire (Table 1).

### 3.5 Healthcare professionals

Most healthcare professionals were aged 25–36 (44.1%, *n* = 40, *p* = 0.008). The sex ratio was balanced, with a slight predominance of females (58.8%, *n* = 47, *p* = 0,329). Specialist physicians were the most represented (68.5%, *n* = 63) and nearly all worked almost exclusively in a university hospital (97.8%, *n* = 90). Kidney transplantation was the most common type of transplant (55.4%, *n* = 51, *p* = 0.006) (Table 2).

**Table 2:** Characteristics of healthcare professionals involved in solid organtransplantation (SOT) by type of profession, with between-group statistical analysis.

	Coordination nurse (*N* = 5)	Medical resident (*N* = 5)	Pharmacist (*N* = 2)	Pharmacy resident (*N* = 11)	Specialist physician (*N* = 63)	Surgeon (*N* = 6)	Total (*N* = 92)	*p* value
**Sex**	**Sex**	**Sex**	**Sex**	**Sex**	**Sex**	**Sex**	**Sex**	0.3291
Male, *n* (%)	1 (33.3%)	1 (20.0%)	1 (50.0%)	2 (18.2%)	25 (45.5%)	3 (75.0%)	33 (41.2%)	
**Age**	**Age**	**Age**	**Age**	**Age**	**Age**	**Age**	**Age**	0.008
18–25, *n* (%)	0 (0.0)	0 (0.0)	0 (0.0)	2 (18.2)	0 (0.0)	0 (0.0)	2 (2.4)	
26–35, *n* (%)	4 (80.0)	1 (20.0)	5 (100.0)	9 (81.8)	20 (35.1)	1 (50.0)	40 (47.1)	
36–50, *n* (%)	0 (0.0)	2 (40.0)	0 (0.0)	0 (0.0)	24 (42.1)	1 (50.0)	27 (31.8)	
51–65, *n* (%)	1 (20.0)	2 (40.0)	0 (0.0)	0 (0.0)	10 (17.5)	0 (0.0)	13 (15.3)	
> 65, *n* (%)	0 (0.0)	0 (0.0)	0 (0.0)	0 (0.0)	3 (5.3)	0 (0.0)	3 (3.5)	
**Organ**	**Organ**	**Organ**	**Organ**	**Organ**	**Organ**	**Organ**	**Organ**	0.0061
Liver, *n* (%)	0 (0%)	1 (20%)	0 (0%)	2 (18.2%)	9 (14.3%)	3 (50%)	15 (16.3%)	
Heart, *n* (%)	0 (0%)	0 (0%)	0 (0%)	0 (0%)	5 (7.9%)	0 (0%)	5 (5.4%)	
Kidney, *n* (%)	3 (60%)	4 (80%)	0 (0%)	1 (9.1%)	40 (63.5%)	3 (50%)	51 (55.4%)	
Lung, *n* (%)	1 (20%)	0 (0%)	1 (50%)	6 (54.5%)	5 (7.9%)	0 (0%)	13 (14%)	
**I know about AI** ^a^	**I know about AI** ^a^	**I know about AI** ^a^	**I know about AI** ^a^	**I know about AI** ^a^	**I know about AI** ^a^	**I know about AI** ^a^	**I know about AI** ^a^	0.9731
Yes (%)	5/5 (100%)	5/5 (100%)	2/2 (100%)	5/5 (100%)	46/48 (96%)	3/3 (100%)	66/68 (97%)	
**Context of declared knowledge on AI**	**Context of declared knowledge on AI**	**Context of declared knowledge on AI**	**Context of declared knowledge on AI**	**Context of declared knowledge on AI**	**Context of declared knowledge on AI**	**Context of declared knowledge on AI**	**Context of declared knowledge on AI**	0.192
Media, *n* (%)	1 (20%)	2 (40%)	0 (0%)	0 (0%)	6 (10%)	0 (0%)	10 (12.7%)	
Professional, *n* (%)	1 (20%)	1 (20%)	1 (50%)	6 (70%)	23 (40%)	3 (80%)	36 (45.6%)	
Medical, n(%)	0 (0%)	0 (0%)	0 (0%)	0 (0%)	6 (10%)	1 (20%)	7 (8.9%)	
**AI declared declared knowledge**	**AI declared declared knowledge**	**AI declared declared knowledge**	**AI declared declared knowledge**	**AI declared declared knowledge**	**AI declared declared knowledge**	**AI declared declared knowledge**	**AI declared declared knowledge**	0.0412
Mean (SD)	4 (2.5)	3.2 (2.5)	3.5 (2.1)	2.3 (2.3)	4 (1.7)	6 (1.2)	3.9 (2)	
**I know about AI on healthcare** ^a^	**I know about AI on healthcare** ^a^	**I know about AI on healthcare** ^a^	**I know about AI on healthcare** ^a^	**I know about AI on healthcare** ^a^	**I know about AI on healthcare** ^a^	**I know about AI on healthcare** ^a^	**I know about AI on healthcare** ^a^	0.5901
Yes (%)	3/4 (75%)	5/5 (100%)	2/2 (100%)	9/9 (100%)	48/52 (92.3%)	4/4 (100%)	71/76 (93.4%)	
**AI on healthcare declared declared knowledge**	**AI on healthcare declared declared knowledge**	**AI on healthcare declared declared knowledge**	**AI on healthcare declared declared knowledge**	**AI on healthcare declared declared knowledge**	**AI on healthcare declared declared knowledge**	**AI on healthcare declared declared knowledge**	**AI on healthcare declared declared knowledge**	0.0652
Mean (SD)	3 (3.6)	2.8 (1.8)	3 (1.4)	1.9 (2.4)	3.5 (1.9)	5.8 (1)	3.3 (2.1)	
**I know about AI on SOT** ^a^	**I know about AI on SOT** ^a^	**I know about AI on SOT** ^a^	**I know about AI on SOT** ^a^	**I know about AI on SOT** ^a^	**I know about AI on SOT** ^a^	**I know about AI on SOT** ^a^	**I know about AI on SOT** ^a^	0.581
Yes (%)	3/4 (75%)	3/5 (60%)	1/2 (50%)	1/9 (11.1%)	27/52 (51.9%)	4/4 (100%)	39/76 (51.3%)	
**AI on SOT declared declared knowledge**	**AI on SOT declared declared knowledge**	**AI on SOT declared declared knowledge**	**AI on SOT declared declared knowledge**	**AI on SOT declared declared knowledge**	**AI on SOT declared declared knowledge**	**AI on SOT declared declared knowledge**	**AI on SOT declared declared knowledge**	0.172
Mean (SD)	2.8 (3.2)	1.4 (1.7)	1 (1.4)	0.9 (2.7)	2.6 (2.5)	6.2 (1.7)	2.5 (2.6)	
**Use of AI**	**Use of AI**	**Use of AI**	**Use of AI**	**Use of AI**	**Use of AI**	**Use of AI**	**Use of AI**	0.4481
Yes, *n* (%)	2 (50%)	0 (0%)	1 (50%)	1 (11.1%)	15 (28.3%)	1 (25%)	20 (26%)	
**AI opinion**	**AI opinion**	**AI opinion**	**AI opinion**	**AI opinion**	**AI opinion**	**AI opinion**	**AI opinion**	0.3662
Mean (SD)	6.5 (1)	6 (1.4)	6 (1.4)	5.6 (1)	6.2 (1.8)	7.8 (0.5)	6.2 (1.6)	
**Most popular application**	**Most popular application**	**Most popular application**	**Most popular application**	**Most popular application**	**Most popular application**	**Most popular application**	**Most popular application**	0.5931
Donor-patient matching, *n* (%)	2 (50%)	3 (60%)	0 (0%)	4 (44.4%)	19 (36.5%)	3 (75%)	31 (40.8%)	
Medical imagery analysis, *n* (%)	1 (25%)	0 (0%)	0 (0%)	1 (11.1%)	6 (11.5%)	1 (25%)	9 (11.8%)	
Morbi-mortality prediction, *n* (%)	0 (0%)	2 (40%)	1 (50%)	1 (11.1%)	18 (34.6%)	0 (0%)	22 (28.9%)	
Therapeutical follow up, *n* (%)	1 (25%)	0 (0%)	1 (50%)	3 (33.3%)	9 (17.3%)	0 (0%)	14 (18.4%)	
**Find out more about AI**	**Find out more about AI**	**Find out more about AI**	**Find out more about AI**	**Find out more about AI**	**Find out more about AI**	**Find out more about AI**	**Find out more about AI**	0.1201
Yes, *n* (%)	3.0 (75.0%)	3.0 (75%)	2.0 (100%)	6.0 (66.7%)	19.0 (44%)	4 (100%)	37 (56.1%)	

1 Pearson's Chi-squared test.

2 Linear Model ANOVA.

a n/ n of patients who responded to the item.

In cases where data was missing, this was specified for each item concerned.

### 3.6 Artificial intelligence declared knowledge

Almost all healthcare professionals had already heard of AI (97.1%, *n* = 66), frequently through their professional environment. ADL of knowledge was higher than that of patients (mean=3.8/10) (Supplementary Table S3). The most used AI was a diagnostic algorithm and a chatbot. Most had heard of AI in healthcare (93.4%, *n* = 71) with a low ADL of knowledge (mean =3.3/10) (Table 2). Half had heard of the application of AI in SOT (51.3%, *n* = 39), with a very low ADL of knowledge (mean = 2.4/10). 26.0% (*n* = 20) had already experimented with AI at work with moderate helpfulness (mean = 5.3/10), mainly for mortality prediction scores, immunosuppressant dose monitoring, and graft allocation.

### 3.7 Artificial intelligence perception

The perception of AI by healthcare professionals was rather positive (6.2/10) (Table 2). Overall, 108 different words were mentioned; the most frequent were “help” (24.5%, *n* = 23), “future” (16.0%, *n* = 15), “rapidity” (14.9%, *n* = 14), and “robots” (12.8%, *n* = 12), i.e., positive-sounding words. Five themes emerged: emotion, applications, negative points, positive points, and method. All emotions were negative, but the positive point category was the most represented. ChatGPT was the first application mentioned. The most popular application of AI in SOT was graft allocation (40.8%, *n* = 31) (Table 2).

The main risk perceived was the loss of reasoning, i.e., “forget to think” (14.9%, *n* = 14), followed by dehumanization and AI errors at equality (10.6%, *n* = 10). Main opportunities were timesaving and error reduction equally (11.7%, *n* = 13). Several applications were mentioned, with better donor-recipient matching being the most cited. A significant proportion had no opinion on these two questions (22.5%, *n* = 21). Half of the healthcare professionals expressed interest in learning more about AI after completing the questionnaire (Table 2).

### 3.8 Comparison between populations

Transplanted organ type was not associated with the level of declared knowledge about AI and patient perception. Perception was positively associated with the declared level of knowledge about AI (regression coefficient: 0.265; 95% CI: 0.10; 0.43; *p* = 0.005). Perception was not associated with the level of declared knowledge of AI in healthcare (*p* = 0.270) and in SOT. (*p* = 0.392) (Supplementary Table S1). The type of organ transplanted was associated with a different level of declared knowledge (Table 2).

Surgeons have a significantly higher level of declared knowledge than other professions for AI in SOT (regression coefficient: 3.70; 95% CI: 1.04, 6.36; *p* = 0.006) (Supplementary Table S2). The perception of AI by healthcare professionals was associated with the level of knowledge on global and SOT AI (Supplementary Table S2).

Healthcare professionals had heard more about AI than patients at the health and transplant levels, especially for the use of AI in healthcare (*p* < 0.001) (Supplementary Table S3). Similarly, healthcare professionals had a declared higher level of knowledge of AI than patients at both levels. There was no difference in the perception of AI in SOT between the two groups. Declared knowledge was statistically and positively associated with perception of AI (Supplementary Table S3).

## 4 Discussion

This study was exploratory and aimed to provide a first assessment of perceptions and declared knowledge regarding AI in SOT. It highlighted a very low level of declared knowledge for both studied groups, particularly among patients. The gap between patients and healthcare professionals is probably multifactorial. Healthcare professionals are regularly exposed to clinical innovation and continuing education, including emerging technologies like AI, contributing to a better baseline understanding. These results suggest that the level of declared knowledge may influence perception of AI, which appears to be associated with more cautious attitudes toward AI use in clinical care. This aligns with prior research suggesting that insufficient understanding contributes to mistrust in the healthcare context (18). Moreover, qualitative analysis of patients' responses suggested that they perceived AI as assistance to the medical profession. Several studies have shown that patients, despite a positive perception, will always favor their physician's opinion if the results are discordant (19–21). The most commonly perceived risks included the risk of error and dehumanization, in line with other studies suggesting that individuals are likely to suggest a lack of confidence in the features of AI systems (22–24). As trust is a key factor in the adoption of AI tools (25), our findings underscore the importance of maintaining the healthcare professional at the center of decision-making, with AI used as support rather than replacement. This nuanced perception suggests opportunities to design AI tools with embedded roles for shared decision-making and transparent communication. However, a recent study comparing perceptions of AI-generated responses with those of the National Kidney Foundation suggested that participants preferred the AI-generated responses in 81.3% of cases in terms of information quality, empathy, and perceived learning (26). These findings suggest that generative AI has the potential to deliver information in a manner that is appropriate for patients.

Healthcare professionals play a central role in the acceptability and integration of AI in SOT as they are responsible for evaluating, implementing, and monitoring AI systems in clinical practices. They also have a decisive role to play in educating patients about the use of AI in their healthcare, which may improve patients' acceptance and understanding. Therefore, close multidisciplinary collaboration is crucial to integrate AI into transplant patient care processes and improve acceptability.

Our results are relevant to all actors involved in the development and/or use of AI in medicine. Targeted strategies are needed to guide AI adoption among healthcare professionals and patients (27).. However, ethical consideration and data privacy standards must be respected to ensure the security and responsible use of health data. Ethical challenges include the need for transparency, accountability, and fairness in algorithmic decision-making processes. In high-stakes fields such as SOT, AI tools must be interpretable to ensure that clinicians do not rely on them all uncritically and patients can remain active participants in their care. Bias in training datasets poses a risk of exacerbating existing health disparities, disproportionately affecting vulnerable populations. Concerns around dehumanization also raise questions about preserving patient autonomy and relational trust. Considering these ethical and societal dimensions is essential for both regulatory compliance and long-term acceptability. Our findings align with the broader European Union's AI strategy, which emphasizes the development of trustworthy, human-centric AI (28). The 2021 Coordinated Plan on Artificial Intelligence aims to foster AI that respects fundamental rights, enhances healthcare delivery, and involves citizens in its design and deployment. By identifying gaps previously cited, our study supports inclusive development processes that integrate end users early, thereby enhancing acceptance and alignment with ethical principles.

### 4.1 Limitations

This study has several limitations. The low response rate to the questionnaire in relation to the number of patients transplanted in France limits the statistical power, but we identified important elements and used various recruitment channels. The distribution of the type of organ transplanted in the questionnaire responses was not representative of the national population. While kidney transplantation represents approximately 60% of SOT procedures annually in France and 70% worldwide, only 8.7% of patient respondents had received a kidney transplant, due to the limited distribution of the questionnaire among this population (29). This underrepresentation may reflect lower digital engagement among older kidney transplant recipients, as most of the responses were online (30). Furthermore, as kidney transplant patients tend to be older, the reported levels of declared knowledge may well have been even lower. As the type of organ transplanted was not associated with the level of declared knowledge or perception, we suggest that this bias did not have an impact on the results. Very few patients over the age of 65 completed the questionnaire. This is because the retirement age in France is around 60–65, meaning that only a small proportion of professionals continue to work beyond the age of 65. Moreover, recruitment via transplant patient associations may have introduced a selection bias, as these patients are probably more involved and informed, which could lead to an overestimation of the level of declared knowledge. These results should therefore be treated with caution in terms of generalizability.

Although semi-structured interviews and focus groups might have provided deeper insights, they also present limitations in terms of subjectivity and the required time. Regarding qualitative analysis, the subjectivity of the interpretation was limited by the fact that two people analyzed the responses independently. Sole reliance on self-assessed declared knowledge without objective measures may bias results, too. Demographic data were limited, and response rate per group was not always available, reducing the ability to fully assess the representativeness of the sample.

A limitation of this study relates to the level of granularity of the AI concepts assessed in the questionnaire. AI in healthcare and especially in transplantation has many types, including machine learning, predictive analytics, and clinical decision support systems, as well as a wide range of clinical applications. However, our survey did not differentiate between these specific categories. This choice was made to preserve accessibility and ensure comprehension across heterogeneous respondent groups, including patients and non-specialist healthcare professionals, for whom a more detailed stratification of AI types and applications may have introduced excessive complexity and potential misunderstanding. This limitation is consistent with the current literature on perceptions of artificial intelligence in healthcare, where most survey-based studies assess AI as a broad, non-specific concept rather than distinguishing between distinct technical subfields or clinical applications (26, 31)Furthermore, the internal consistency of the questionnaire was poor: Cronbach's alpha coefficient was low for patients and confidence interval for healthcare professionals was wide However, prior to distribution, the questionnaire was reviewed by experts in the humanities and social sciences, transplant clinicians, and patient associations representing transplant recipients to enhance its clarity, relevance, and overall consistency. This may reflect variability in patients' understanding of questionnaire items, probably influenced by limited prior exposure to AI concepts, social desirability bias, or the self-administered nature of the survey (32). The halo effect should not be overlooked either, as respondents may be influenced by a previous question or overall impressions (33). Although there is no external benchmark for AI knowledge scores in this context, we provide an interpretation by analyzing relations between mean scores and participants' qualitative responses and observed patterns, offering insight into their declared knowledge and information gaps. Finally, the exclusion of patients' family members, who often play an important role in decision-making and care management, represents a future opportunity to explore broader perspectives that could influence the acceptability and implementation of AI. Consequently, results should be interpreted as exploratory and hypothesis-generating rather than confirmatory.

In conclusion, this is the first study investigating solid organ transplanted patients and healthcare professionals on the use of AI for SOT. This study suggested a low level of declared knowledge but a positive perception of AI for both patients and healthcare professionals. Considering the exponential development of AI applications in SOT, these results should encourage government authorities and educational establishments to better inform patients and healthcare professionals about the use of AI in SOT.

## Data Availability

The raw data supporting the conclusions of this article will be made available by the authors, without undue reservation.
