# Supplementary material for: Assessing knowledge and perception of artificial intelligence in solid organ transplantation
Source: Front Digit Health. 2026 Jul 8;8:1834577. doi: 10.3389/fdgth.2026.1834577 (PMC13388862; doi:10.3389/fdgth.2026.1834577)
Supplement: Supplementary file 1 [file Supplementaryfile1.docx]

Supplementary Material

# Supplementary Data

## Survey characteristics:

### Survey construction

The main themes for the questionnaire were identified through a literature review on the application of AI to SOT, using PubMed, Embase, and Cochrane databases. The search strategy included a combination of free-text terms and MeSH terms related to AI, SOT, cross-sectional studies, knowledge, and perception. This literature review identified examples of AI applications in SOT and how AI can be used. It also provided a basis for constructing items that would enable perception and knowledge to be properly explored (1–4).

The questionnaire included 24 items for patients and 26 items for professionals, divided into 3 categories: socio-demographic (items 1-7 for patients, 1-6 for professionals) , self-assessment of knowledge of AI (items 8-17 excluding 12 and 15 for patients, items 7-10, 12-13, 15-18 for professionals) and perception of AI (items 12,15, 17-24 for patients, items 11,14 and 19-26 for professionals),. For the level of knowledge and perception on AI a numerical scale was used, ranging from 0 to 10. Knowledge on AI was defined by weak (<5), consistent (5-6) and high (>7) level of knowledge.

As part of the evaluation of perception, a Likert scale was used, with respondents expressing their degree of agreement or disagreement on the item “I think the use of artificial intelligence to”.

A qualitative analysis was also carried out using 3 open questions : "What do you think would be the risks of artificial intelligence in the field of transplantation?", "What do you think would be the opportunities offered by artificial intelligence in the field of transplantation?" and "Give 5 words that come to mind when you think of artificial intelligence".

The questionnaire was drafted in French and administered to French patients and healthcare professionals, so did not need to be translated.

### Patient survey

1. **You are:**
□ Male
□ Female

□ None

2. **Which age group do you belong to?**
□ 18–25 years
□ 26–35 years
□ 36–50 years
□ 51–65 years
□ 65 years and older

3. **What is your department of residence ?**
(Dropdown menu of departments if online)

4. **What type of profession do you practice ?**

1. Farmer
2. Artisan
3. Shopkeeper
4. Business owner
5. Self-employed professional
6. Administrative and technical managers in the public sector
7. Administrative and commercial managers in the private sector
8. Higher education teacher
9. Engineer or technical manager in the private sector
10. Primary and vocational school teacher
11. Health and social work intermediary professionals
12. Clergy
13. Public sector intermediary professionals (administration, security)
14. Administrative and commercial intermediary professionals in companies
15. Technicians
16. Supervisors
17. Employees
18. Workers
19. Vehicle drivers (transportation)
20. Retired
21. Unemployed

5. **How many transplants have you undergone?**
□ 1
□ 2
□ 3
□ More than 3

6. **What type(s) of organ(s) were transplanted ?**
□ Heart
□ Liver
□ Lung
□ Kidney

7. **In what year did you have your most recent transplant ?**
………………………………………………………………………………

8. **Have you heard of artificial intelligence (AI) ?**
□ Yes
□ No

9. **If yes, in what context have you heard about artificial intelligence ?**
□ Professional
□ Medical
□ Societal
□ Media
□ Other (if other, specify: …………………………)

10. **How would you evaluate your knowledge of this topic ?**

11. **What type(s) of artificial intelligence algorithms do you know ?**
□ Medical diagnosis support algorithms
□ Machine learning (supervised or unsupervised)
□ Deep learning (image recognition or natural language processing)
□ Conversational agents or chatbots
□ Other (if other, specify: …………………………)
□ None

12. **List 5 words that come to mind when you think about artificial intelligence.**

13. **Have you heard of artificial intelligence in healthcare ?**
□ Yes
□ No

14. **How would you evaluate your knowledge of this topic ?**

15. **To what extent do you agree with the following statements ?**

- AI will replace doctors for conducting and evaluating medical exams. □ Yes □ No
- AI performs better than humans. □ Yes □ No
- AI scares me. □ Yes □ No
- AI is the future of healthcare. □ Yes □ No
- To produce new innovative drugs, pharmaceutical companies should feed their AI algorithms with patient medical data. □ Yes □ No
- The use of AI requires full protection of health data. □ Yes □ No

16. **Have you heard of artificial intelligence in the field of transplantation ?**
□ Yes
□ No

17. **How would you evaluate your knowledge of this topic ?**

18. **Below are several applications of AI in transplantation. Please rank them in order of interest to you (1 = most important):**
□ Prediction model for morbidity and mortality (risk of mortality or complications related to diseases)
□ Donor-recipient matching (genetic and viral compatibility between donor and recipient)
□ Imaging interpretation (scans, radiology, MRI usually performed by a doctor)
□ Therapeutic pharmacological monitoring (regular blood level monitoring of immunosuppressants or anti-infective agents)

19. **On a scale of 1 to 10, what is your opinion on artificial intelligence ?**

20. **What do you think are the risks of artificial intelligence in transplantation ?**

21. **What do you think are the opportunities offered by artificial intelligence in transplantation ?**

22. **I think the use of artificial intelligence to:**

| **Statement** | **A benefit** | **A violation of patient rights** | **A lack of ethics** | **An inequity for certain patients** | **Exploitation of my medical data for other purposes** | **Neutral opinion** |
| --- | --- | --- | --- | --- | --- | --- |
| Improve the diagnosis of transplant complications (e.g., infections) is... | □ | □ | □ | □ | □ | □ |
| Better predict the risk of rejection is... | □ | □ | □ | □ | □ | □ |
| Develop new drugs is... | □ | □ | □ | □ | □ | □ |
| Optimize monitoring of immunosuppressants (blood concentrations) is... | □ | □ | □ | □ | □ | □ |
| Enhance adverse effect monitoring is... | □ | □ | □ | □ | □ | □ |

23. **What would you be willing to share with artificial intelligence for your care?**
□ All necessary data to receive the best possible care
□ Basic data only (age, gender, region, etc.)
□ Very little data, as I have little trust in AI

24. **Will you seek more information about artificial intelligence after completing this questionnaire?**
□ Yes
□ No

### Healthcare professional survey

**1. You are...**
□ Male
□ Female

□ None

**2. What age group do you belong to?**
□ 18–25 years
□ 26–35 years
□ 36–50 years
□ 51–65 years
□ 65 years and older

**3. What is your department of residence?**
…………………………………………….

**4. What is your occupation?**
□ Surgeon
□ Specialist physician
□ Coordination nurse
□ Pharmacist
□ Medical resident
□ Pharmacy resident
□ General practitioner
□ Other
If other, please specify: ………………

**5. What type of facility do you work in?**
□ University hospital
□ General hospital
□ Non-profit clinic
□ For-profit clinic
□ Rehabilitation or post-acute care facility
□ Other
If other, please specify: ………………

**6. What type(s) of solid organ transplant are you specialized in?**
□ Heart
□ Liver
□ Lung
□ Kidney

**7. Have you heard of artificial intelligence?**
□ Yes
□ No

**8. In what context have you heard about artificial intelligence?**
□ Professional
□ Medical
□ Societal
□ Media
□ Other (if other, specify the context: …………………)

**9. How would you assess your knowledge on the subject?**
…………………………………………………..

**10. What type(s) of artificial intelligence algorithms do you know?**
□ Medical diagnostic assistance algorithms
□ Machine learning (supervised or unsupervised statistical models)
□ Deep learning (image recognition or natural language processing)
□ Conversational agents or chatbots
□ Other (if other, specify: …………………)
□ None

**11. List five words that come to mind when you think of artificial intelligence.**
…………………………………………………..

**12. Have you heard about artificial intelligence in healthcare?**
□ Yes
□ No

**13. How would you assess your knowledge on this topic?**
…………………………………………………..

**14. To what extent do you agree with the following statements?**

- Artificial intelligence will replace physicians in performing and interpreting medical exams.
  □ Yes
  □ No
- Artificial intelligence performs better than a human.
  □ Yes
  □ No
- Artificial intelligence scares me.
  □ Yes
  □ No
- Artificial intelligence is the future of healthcare.
  □ Yes
  □ No
- To develop a new innovative drug, pharmaceutical companies should feed artificial intelligence algorithms with patients' medical data.
  □ Yes
  □ No
- The use of artificial intelligence requires full protection of health data.
  □ Yes
  □ No

**15. Have you heard about artificial intelligence in solid organ transplantation?**
□ Yes
□ No

**16. How would you assess your knowledge of the subject?**
…………………………………………………..

**17. Have you ever used artificial intelligence in your practice?**
□ Yes
□ No

**18. If yes, what type(s) of artificial intelligence have you used and in what context(s)/purpose(s)?**
…………………………………………………..

**19. If yes, on a scale of 1 to 10, how useful did you find it?**…………………………………………………..

**20. Below are several types of artificial intelligence applications in transplantation. Please rank them by your level of interest (1 = most important):**
□ Morbidity and mortality prediction models (e.g., predicting mortality risk or complications from diseases)
□ Donor-recipient matching (genetic and viral compatibility between donor and recipient)
□ Image analysis (CT scans, radiology, MRI normally interpreted by physicians)
□ Pharmacological therapeutic monitoring (regular blood testing of immunosuppressants or anti-infective agents)

**21. On a scale of 1 to 10, what is your opinion on artificial intelligence?**
…………………………………………………..

**22. What do you see as the risks of artificial intelligence in the field of solid organ transplantation?**
…………………………………………………..

**23. What opportunities do you see with artificial intelligence in the field of solid organ transplantation?**
…………………………………………………..

**24. To what extent do you agree with the following statements?**

- Artificial intelligence will replace physicians in performing and interpreting medical exams.
  □ Yes
  □ No
- Artificial intelligence performs better than a human.
  □ Yes
  □ No
- Artificial intelligence scares me.
  □ Yes
  □ No
- Artificial intelligence is the future of healthcare.
  □ Yes
  □ No
- To develop a new innovative drug, pharmaceutical companies should feed artificial intelligence algorithms with patients' medical data.
  □ Yes
  □ No

**25. I think the use of artificial intelligence to:**

| **Statement** | **A benefit** | **A violation of patient rights** | **A lack of ethics** | **An inequity for certain patients** | **Exploitation of my medical data for other purposes** | **Neutral opinion** |
| --- | --- | --- | --- | --- | --- | --- |
| Improve the diagnosis of transplant complications (e.g., infections) is... | □ | □ | □ | □ | □ | □ |
| Better predict the risk of rejection is... | □ | □ | □ | □ | □ | □ |
| Develop new drugs is... | □ | □ | □ | □ | □ | □ |
| Optimize monitoring of immunosuppressants (blood concentrations) is... | □ | □ | □ | □ | □ | □ |
| Enhance adverse effect monitoring is... | □ | □ | □ | □ | □ | □ |

**26. Will you seek more information on artificial intelligence after completing this questionnaire?**
□ Yes
□ No

### Ethical consideration

This study was conducted in accordance with ethical principles of the Declaration of Helsinki. Prior to participation, all patients were provided with detailed information about the study, including its objectives, procedures, potential risks, and benefits. A non-objection form was provided to them. To maintain confidentiality, all responses were anonymized and securely stored in compliance with data protection regulation. Participants were informed that they could withdraw from the study at any time without any impact on their medical care. Ethical approval was obtained from the Personal Information Protection Commission (PCP) Ouest IV with a reference number of 2023-A00950-45.

No personal or identifying information was linked to the survey responses, and all data were used exclusively for the purposes of this research.

# Supplementary Tables

### Table S1: Comparison of Knowledge and Perception Among Solid Organ Transplanted Patients. Between-group comparisons were performed using linear regression and estimate the Regression coefficients and its 95% confidence interval

|  | **Regression Coefficients** | **95% confidence interval** | **p-value** |
| --- | --- | --- | --- |
| **AI declared knowledge** | | | 0.321 |
| Liver* | 2.38 | 1.95 ;2.81 |  |
| Kidney | 0.15 | -1.06 ;1.37 | 0.906 |
| Heart | 0.88 | -0.07 ;1.83 | 0.28 |
| Lung | -0.61 | -1.53 ;0.29 | 0.392 |
| **Healthcare AI declared knowledge** | | | 0.476 |
| Liver* | 1.27 | 0.91 ;1.64 |  |
| Kidney | 0.09 | -0.97 ;1.15 | 0.925 |
| Heart | 0.64 | -0.20 ;1.49 | 0.368 |
| Lung | -0.41 | -1.19 ;0.37 | 0.518 |
| **Transplantation AI declared knowledge** | | | 0.495 |
| Liver* | 0.39 | 0.17 ;0.61 |  |
| Kidney | 0.21 | -0.40 ;0.83 | 0.0.676 |
| Heart | 0.47 | -0.01 ;0.95 | 0.270 |
| Lung | 0.04 | -0.43 ;0.52 | 0.925 |
| **AI perception** | | | 0.582 |
| AI declared knowledge | 0.265 | 0.10 ;0.43 | 0.005 |
| AI applicated to healthcare declared knowledge | 0.193 | 0.0 ;0.39 | 0.270 |
| AI applicated to transplantation declared knowledge | 0.217 | -0.10 ;0.54 | 0.392 |

* Represents the reference level.

AI: artificial intelligence

### Table S2: Comparison of Knowledge and Perception Among Solid Organ Transplanted Healthcare professionals. Between-group comparisons were performed using linear regression and estimate the Regression coefficients and its 95% confidence interval

|  | **Regression Coefficients** | **95% confidence interval** | **p-value** |
| --- | --- | --- | --- |
| **AI declared knowledge** | | | 0.270 |
| Specialist physician* | 2.37 | -1.8 ; 6.54 |  |
| Coordination nurse | -0.01 | -1.82 ; 1.79 | 0.989 |
| Medical resident | -0.67 | -2.62 ; 1.28 | 0.676 |
| Pharmacist | -0.55 | -3.33 ; 2.23 | 0.833 |
| Pharmacy resident | -1.37 | -3.01 ; 0.27 | 0.321 |
| Surgeon | 2.04 | 0.04 ; 4.05 | 0.207 |
| **Healthcare AI declared knowledge** | | | 0.270 |
| Specialist physician* | 1.21 | -3.27 ; 5.69 |  |
| Coordination nurse | -0.50 | -2.64 ; 1.64 | 0.797 |
| Medical resident | -0.53 | -2.63 ; 1.56 | 0.788 |
| Pharmacist | -0.46 | -3.44 ; 2.53 | 0.885 |
| Pharmacy resident | -1.21 | -2.97 ; 0.55 | 0.392 |
| Surgeon | 2.31 | 0.16 ; 4.46 | 0.172 |
| **Transplantation AI declared knowledge** | | | 0.392 |
| Specialist physician* | 1.26 | -4.27 ; 6.80 |  |
| Coordination nurse | 0.08 | -2.56 ; 2.72 | 0.980 |
| Medical resident | -0.86 | -3.45 ; 1.72 | 0.676 |
| Pharmacist | -1.50 | -5.19 ; 2.19 | 0.629 |
| Pharmacy resident | -1.26 | -3.44 ; 0.91 | 0.476 |
| Surgeon | 3.70 | 1.04 ; 6.36 | 0.0064 |
| **AI perception** | | | 0.582 |
| AI declared knowledge | 0.3 | 0.10 ; 0.50 | 0.005 |
| AI applicated to healthcare declared knowledge | 0.21 | 0.0 ; 0.42 | 0.270 |
| AI applicated to transplantation declared knowledge | 0.23 | 0.08 ; 0.39 | 0.048 |

* Represents the reference level.

AI: artificial intelligence

### Table S3: Comparison of Knowledge and Perception between Solid Organ Transplanted Patients and Healthcare professionals. Between-group comparisons were performed using linear regression and estimate the Regression coefficients and its 95% confidence interval

|  | **Regression Coefficients** | **95% confidence interval** | **p-value** |
| --- | --- | --- | --- |
| AI declared knowledge | 1.35 | 0.63 ; 2.07 | <0.001 |
| Know about AI | 12.10 | 1.46 ; 101 | 0.138 |
| AI applicated to healthcare declared knowledge | 2.15 | 1.49 ; 2.81 | <0.001 |
| Know about AI applicated to healthcare | 22.00 | 7.12 ; 67.94 | <0.001 |
| AI applicated to transplantation declared knowledge | 2.33 | 1.75 ; 2.92 | <0.001 |
| Know about AI applicated to transplantation | 10.30 | 4.23 ; 25.3 | <0.001 |
| AI perception | 0.67 | -0.15 ;1.48 | 0.107 |

* Represents the reference level.

AI: artificial intelligence

**References**

1. Castagno S, Khalifa M. Perceptions of Artificial Intelligence Among Healthcare Staff: A Qualitative Survey Study. Front Artif Intell. 21 oct 2020;3. doi:10.3389/frai.2020.578983

2. Fritsch SJ, Blankenheim A, Wahl A, Hetfeld P, Maassen O, Deffge S, et al. Attitudes and perception of artificial intelligence in healthcare: A cross-sectional survey among patients. Digit Health. 2022;8:20552076221116772. doi:10.1177/20552076221116772 PubMed PMID: 35983102; PubMed Central PMCID: PMC9380417.

3. Laï MC, Brian M, Mamzer MF. Perceptions of artificial intelligence in healthcare: findings from a qualitative survey study among actors in France. Journal of Translational Medicine. 9 janv 2020;18(1):14. doi:10.1186/s12967-019-02204-y

4. Maassen O, Fritsch S, Palm J, Deffge S, Kunze J, Marx G, et al. Future Medical Artificial Intelligence Application Requirements and Expectations of Physicians in German University Hospitals: Web-Based Survey. Journal of Medical Internet Research. 5 mars 2021;23(3):e26646. doi:10.2196/26646
